# Prevalence and Characteristics of Prediabetes and Metabolic Syndrome in Seemingly Healthy Persons at a Health Check-Up Clinic

**DOI:** 10.2147/JMDH.S374164

**Published:** 2022-07-23

**Authors:** Watip Tangjittipokin, Lanraphat Srisawat, Nipaporn Teerawattanapong, Tassanee Narkdontri, Mayuree Homsanit, Nattachet Plengvidhya

**Affiliations:** 1 Department of Immunology, Faculty of Medicine Siriraj Hospital, Mahidol University, Bangkok, Thailand; 2 Siriraj Center of Research Excellence for Diabetes and Obesity (SiCORE-DO), Faculty of Medicine Siriraj, Mahidol University, Bangkok, Thailand; 3 Research Department, Faculty of Medicine Siriraj Hospital, Mahidol University, Bangkok, Thailand; 4 Department of Preventive and Social Medicine, Faculty of Medicine Siriraj Hospital, Mahidol University, Bangkok, Thailand; 5 Division of Endocrinology and Metabolism, Department of Medicine, Faculty of Medicine Siriraj Hospital, Mahidol University, Bangkok, Thailand

**Keywords:** seemingly healthy persons, health check-up clinic, prediabetes, metabolic syndrome

## Abstract

**Purpose:**

This study investigated the prevalence and characteristics of prediabetes (PreDM) and metabolic syndrome (MetS) in seemingly healthy persons attending a health check-up clinic at a tertiary care hospital.

**Patients and Methods:**

This was a cross-sectional study that enrolled 1213 subjects (339 male, 874 female) who underwent an annual health check-up at Siriraj Hospital, Bangkok, Thailand from 2009 to 2019. Factors that independently related to PreDM were analyzed using unconditional logistic regression analysis with adjustments for age, BMI, and gender.

**Results:**

The prevalence of PreDM and MetS was 54.3% and 19.7% respectively. Participants with impaired fasting glucose (IFG) and glycated hemoglobin (HbA_1c_) 38.8–46.4 mmol/mol had significantly higher waist circumference (WC) and blood pressure (BP) compared to those with IFG or HbA_1c_ 38.8–46.4 mmol/mol alone (*P* < 0.05). Among three PreDM subgroups, the average age was lowest in the HbA_1c_ 38.8–46.4 mmol/mol subgroup (*P* < 0.001). PreDM participants with MetS were older (*p* = 0.03), had higher WC, BP, fasting plasma glucose and serum triglyceride level (all *P* < 0.001) but had lower serum high-density lipoprotein (HDL) cholesterol level (*P* < 0.001). Multivariate analysis revealed high MetS score, obesity, and low serum HDL cholesterol level to be independently associated with PreDM with odds ratios of 9.02 (95% confidence interval [CI]: 4.03–20.18), 1.8 (95% CI: 1.07–3.04), and 1.42 (95% CI: 1.02–1.96), respectively.

**Conclusion:**

The prevalence of PreDM and MetS was relatively high in seemingly healthy persons. Distinct PreDM subgroups with or without MetS exhibited diverse clinical and biochemical features suggesting dissimilar pathogenesis.

## Introduction

Prediabetes (PreDM) is characterized by increased glycemia but at levels lower than those that define diabetes. Approximately 25% of patients with PreDM will progress to overt type 2 diabetes mellitus (T2D) within 3–5 years.^1^ PreDM was also reported to increase the risk of macrovascular diseases^2–4^ and heart failure.^5^,^6^ The US Department of Health and Human Services estimated that about one in four US adults aged 20 years or older (approximately 57 million people) has PreDM.^7^ Several epidemiological studies demonstrated a clear relationship between ethnicity and the likelihood of developing PreDM, with African Americans, Native Americans, South Asians, and Hispanics all having been shown to have an increased risk of having PreDM when compared with their Caucasian counterparts.^1^ In Asia, the prevalence of PreDM in Chinese, Saudi Arabians, Indians, and Malaysians was 35.7%, 6.8%, 6.3%, and 22.6%, respectively.^8^ The prevalence of PreDM and T2D in Thai adults was 5.4% and 9.6%, respectively.^9^

Metabolic syndrome (MetS) comprises a cluster of metabolic abnormalities, including hyperglycemia, dyslipidemia, abdominal/central obesity, and high blood pressure. The risk of having heart disease, stroke, or diabetes was shown to be increased 1.5- to 3-fold in people with MetS compared to those without MetS.^10^ The prevalence of MetS in the general population is estimated to be 20–30%, and its prevalence is increasing. In the United States, the prevalence of MetS was estimated at 23.7%,^11^ while the rate of MetS was 21.9% in Thailand and 49.4% in Malaysia.^11^ The prevalence of MetS in South Korea significantly increased from 24.9% in 1998 to 31.3% in 2007.^12^ The prevalence of MetS was 29.3% among middle-aged Chinese men in mainland China, whereas the prevalence among professional drivers in Hong Kong was 26.8%.^13^ A cohort study conducted in Hong Kong found the crude percentage of MetS to be increased from 9.6% during 1990–1999 to 23.0% during 2000–2009.^14^ These results indisputably demonstrated the role of MetS as a rapidly evolving global health concern.^15^ Previous studies reported that coexisting MetS and PreDM may predict the future development of T2D.^16^ Although MetS and PreDM are strongly interrelated, it is unclear whether they influence the same increased risk for cardiovascular complications.

A factor that complicates the identification of these at-risk individuals is that people with PreDM and/or MetS are usually asymptomatic. A regular health check-up, which includes anthropometrical measurements and essential biochemical assessments, is, therefore, a valuable tool recommended to identify them. In addition, the prevalence of coexisted PreDM and MetS has not been studied in Thai population. The aim of this study was to investigate the prevalence and characteristics of PreDM and MetS in seemingly healthy persons attending a health check-up clinic.

## Materials and Methods

### Study Population

**Figure 1:**
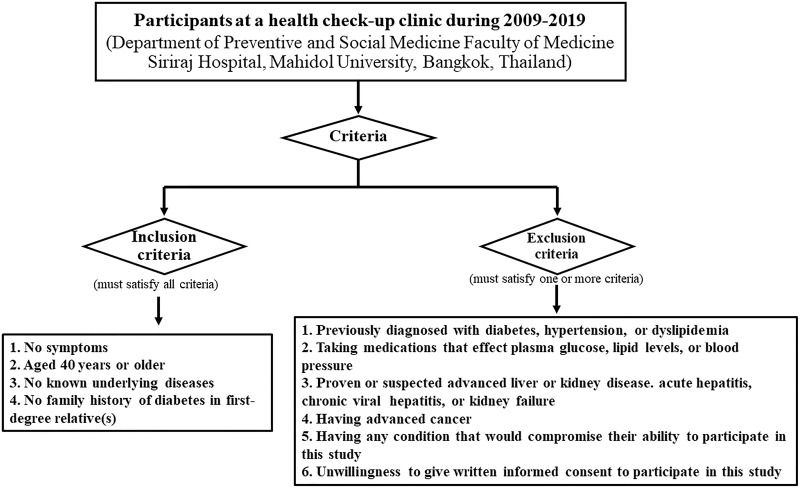
Flow Chart of Study Identification Based on the Inclusion and Exclusion Criteria.

A total of 1213 participants (874 women, 339 men) underwent an annual health check-up at the Department of Preventive and Social Medicine, Faculty of Medicine Siriraj Hospital, Mahidol University, Bangkok, Thailand during 2009–2019 and were prospectively enrolled. Written informed consent was obtained from all participants. The study protocol was approved by the Siriraj Institutional Review Board (SIRB) Faculty of Medicine Siriraj Hospital, Mahidol University (COA no. Si 107/2009 and COA no. Si 491/2014). Participants had to satisfy all of the following inclusion and exclusion criteria (Figure 1).

### Data Collection and Measurements

Data, including age, gender, weight, height, body mass index (BMI), waist circumference (WC), and blood pressure (BP) were collected. BMI was calculated as the ratio of weight in kilograms divided by height in meters squared (kg/m^2^). Age was classified into three quartiles (Q1: Age < 48 years; Q2: Age 48–61 years; Q3: Age>61 years). BMI was categorized into four groups according to the Asian-Pacific cutoff points, as follows: underweight (BMI < 18.5 kg/m^2^), normal weight (BMI 18.5–22.9 kg/m^2^), overweight (BMI 23–24.9 kg/m^2^), and obese (BMI ≥ 25 kg/m^2^). Systolic and diastolic blood pressures were measured using a standard sphygmomanometer with the patient in an upright position after at least 10 minutes of rest. Waist circumference was measured in a standing position with a soft tape midway between the lowest rib and the iliac crest. Blood samples were obtained for biochemical tests. Collection of venous whole blood samples in labeled ethylenediaminetetraacetic acid (EDTA) tubes were used for immediate analysis of fasting plasma glucose (FPG) and HbA_1c_ after an overnight (12 h) fast, and a portion was allowed to clot. FPG was measured by the enzymatic (hexokinase) method. HbA_1c_ was measured by ion-exchange high-performance liquid chromatography (HPLC) using a D-10 Hemoglobin A_1c_ Testing System (Bio-Rad Laboratories, Hercules, CA, USA), which is certified by both the National Glycohemoglobin Standardization Program (NGSP) and the International Federation of Clinical Chemistry and Laboratory Medicine (IFCC). Serum was separated and used for the analysis of the following lipid parameters using the following method: total cholesterol (TC) using the enzymatic colorimetric method; triglycerides (TG) using the enzymatic colorimetric method; high-density lipoprotein (HDL) cholesterol using a homogeneous enzymatic method. Low-density lipoprotein (LDL) cholesterol are estimated by the formula LDL cholesterol = Total cholesterol-[HDL-C + (triglyceride/5)]. All laboratory tests were performed at the Department of Clinical Pathology, Faculty of Medicine Siriraj Hospital, Mahidol University, Bangkok, Thailand.

### PreDM Diagnostic Criteria

1. PreDM was diagnosed according to the American Diabetes Association (ADA) guideline^17^ if one or more of the following parameters are fulfilled:
2. Impaired fasting glucose (IFG): fasting plasma glucose level of 100–125 mg/dL (5.6–6.9 mmol/L).
3. Impaired glucose tolerance (IGT): plasma glucose level of 140–199 mg/dL (7.8–11.0 mmol/L) after 75 g OGTT.
4. HbA_1c_ of 38.8–46.4 mmol/mol.

However, 75 g OGTT was not performed in this study because of its inconvenience in an outpatient setting, it takes 2 hours and requires experienced personnel to perform. Moreover, the 75 g OGTT has poor reproducibility. The cost per test is 290 Thai baht (9.55 USD). In contrast, the cost of FPG and HbA_1c_ assays are 90 Thai baht (2.96 USD) and 180 Thai baht (5.93 USD), respectively. Thus, FPG and HbA_1c_ are the screening tests of choice for PreDM and DM at our health check-up facility. Therefore, patients with IFG, HbA_1c_ 38.8–46.4 mmol/mol, or both were diagnosed as PreDM in our study.

### Metabolic Syndrome (MetS) Diagnostic Criteria

MetS was diagnosed following the National Cholesterol Education Program Adult Treatment Panel – III (NCEP-ATP III) guideline^18^ if 3 or more of the following 5 criteria are met:

1. Abdominal obesity: Waist circumference (WC) > 102 cm in men, and > 88 cm in women. However, World Health Organization-Asian Pacific Region (WHO-APR) criteria for abdominal obesity (WC > 90 cm in men, and > 80 cm in women) were used instead of NCEP-ATP III criteria in this study.
2. Hypertriglyceridemia: serum triglyceride ≥ 150 mg/d (≥ 1.7 mmol/l).
3. Low HDL cholesterol: serum HDL cholesterol < 40 mg/dL (< 1.03 mmol/l) in men, serum HDL-cholesterol < 50 mg/dL (1.29 mmol/l) in women.
4. High blood pressure: BP ≥ 130/85 mmHg.
5. Fasting plasma glucose: FPG ≥ 100 mg/dL (6.1 mmol/l).

### Metabolic Syndrome Score (MetS Score) Diagnostic Criteria

MetS score = 0 (no MetS).

MetS score = 1 (any one of MetS criteria).

MetS score = 2 (any two of MetS criteria).

MetS score = 3 (any three of MetS criteria).

MetS score = 4 (any four of MetS criteria).

MetS score = 5 (all five MetS criteria).

### Statistical Analysis

SPSS Statistics version 22.0 (SPSS, Inc., Chicago, IL, USA) was used to perform all data analyses, and a *P* less than 0.05 was considered to be statistically significant for all tests. Chi-square test or Fisher’s exact test was used to compare categorical data (reported as number and percentage), and Student’s unpaired *t*-test was used to compare the means of 2 independent groups (reported as mean plus/minus standard deviation). Unconditional logistic regression analysis with adjustment for age, BMI, and gender was performed to identify factors independently associated with PreDM. The results were presented as adjusted odds ratio with 95% confidence interval.

## Results

### Baseline Characteristics of Participants

One thousand two hundred and thirteen participants were classified into three groups: normoglycemia, PreDM, and diabetes. Of those, 541 had normoglycemia (78.93% female), 659 had PreDM (66.8% female), and 13 had diabetes (53.8% female). Subjects with PreDM or diabetes were significantly older than those with normoglycemia (average age 56.65 ± 9.37 years vs 59.92 ± 11.31 years vs 52.82 ± 9.31 years; *P* < 0.001 and *P* = 0.01, respectively) (Table 1). PreDM participants had significantly higher BMI, waist circumference, diastolic blood pressure, creatinine level, total cholesterol level, triglyceride level, and LDL cholesterol level, but had significantly lower HDL cholesterol levels compared to normoglycemic participants (all *P* < 0.05). Diabetic participants had the lowest serum creatinine level.

**Table 1:** Demographic, Clinical, and Laboratory Characteristics Compared Among the Normoglycemia, Prediabetes, and Diabetes Groups

Characteristics	Normoglycemia (n = 541)	PreDM (n = 659)	Diabetes (n = 13)	*P*		
				*P*^a^	*P*^b^	*P*^c^
Female gender	427 (78.93%)	440 (66.8%)	7 (53.8%)	**<0.001**	**0.03**	0.33
Age at survey (years)	52.82 ± 9.31	56.65 ± 9.37	59.92 ± 11.31	**<0.001**	**0.01**	0.22
BMI (kg/m^2^)	23.27 ± 0.16	24.02 ± 3.62	24.34 ± 2.41	**<0.001**	0.30	0.75
– Underweight (< 18.5 kg/m^2^)	31 (5.7%)	28 (4.2%)	0 (0.0%)	**<0.001**	0.63	0.90
– Normal weight (18.5–22.9 kg/m^2^)	243 (44.9%)	245 (37.2%)	5 (38.5%)			
– Overweight (23–24.9 kg/m^2^)	131 (24.2%)	140 (21.2%)	3 (23.1%)			
– Obese (≥ 25 kg/m^2^)	136 (25.1%)	246 (37.3%)	5 (38.5%)			
Waist circumference (cm)	82.91 ± 8.94	84.95 ± 9.28	86.96 ± 7.77	**0.001**	0.11	0.44
Systolic BP (mmHg)	118.84 ± 17.51	122.14 ± 16.38	127.08 ± 18.08	0.11	0.09	0.28
Diastolic BP (mmHg)	70.20 ± 11.87	71.27 ± 11.33	68.00 ± 68.00	**<0.001**	0.51	0.30
FPG (mmol/L)	5.01 ± 0.32	5.5 ± 0.49	9.1 ± 3.12	**<0.001**	**<0.001**	**0.001**
HbA_1c_ (mmol/mol)	34.4 ± 2.97	39.70 ± 3.42	55.02 ± 18.94	**<0.001**	**0.002**	**0.01**
Cr (mmol/L)	68.08 ± 15.03	71.62 ± 15.92	66.32 ± 8.84	**<0.001**	0.34	**0.03**
TC (mmol/L)	5.38 ± 0.99	5.59 ± 0.95	5.27 ± 1.03	**<0.001**	0.70	0.23
TG (mmol/L)	0.45 ± 0.67	1.31 ± 0.84	1.39 ± 0.52	**<0.001**	0.14	0.73
HDL (mmol/L)	1.68 ± 0.45	1.58 ± 0.44	1.46 ± 0.36	**<0.001**	0.08	0.30
LDL (mmol/L)	3.19 ± 0.04	3.41 ± 0.89	3.17 ± 1	**<0.001**	0.94	0.33

**Notes**: Data presented as number and percentage or mean plus/minus standard deviation. Bold *P* values indicate statistically significant. ^a^Normoglycemia vs PreDM. ^b^Normoglycemia vs diabetes. ^c^PreDM vs diabetes.

**Abbreviations**: PreDM, prediabetes mellitus; BMI, body mass index; BP, blood pressure; FPG, fasting plasma glucose; HbA_1c_, glycated hemoglobin; Cr, creatinine; TC, total cholesterol; TG, triglycerides; HDL, high-density lipoprotein cholesterol; LDL, low-density lipoprotein cholesterol.

PreDM participants were divided into 3 groups, including Isolated IFG (n = 147), normal FPG with HbA_1c_ 38.8–46.4 mmol/mol (n = 322) and IFG with HbA_1c_ 38.8–46.4 mmol/mol (n = 190). The demographic, clinical characteristics, and laboratory parameters among three PreDM groups were shown in Table 2. Participants with normal FPG and HbA_1c_ 38.8–46.4 mmol/mol were younger and were more female and had the lowest systolic blood pressure and waist circumference. BMI and serum triglyceride levels were lowest in participants with Isolated IFG. Among the three PreDM subgroups, participants with IFG and HbA_1c_ 38.8–46.4 mmol/mol were the oldest, had the highest BMI, waist circumference, systolic and diastolic blood pressures, FPG, and HbA_1c_ levels but had the lowest serum HDL cholesterol level (*P* < 0.05). There was no significant difference in serum total or LDL cholesterol level among the three PreDM subgroups.

**Table 2:** Demographic, Clinical, and Laboratory Characteristics Compared Among the 3 PreDM Subgroups

Characteristics	Isolated IFG (n = 147)	Normal FPG with HbA_1c_ 38.8–46.4 mmol/mol (n = 322)	IFG with HbA_1c_ 38.8–46.4 mmol/mol (n = 190)	*P*		
				*P*^a^	*P*^b^	*P*^c^
Female gender	91 (61.90%)	237 (73.60%)	112 (58.95%)	**0.01**	0.58	**0.001**
Age at survey (years)	58.41 ± 9.50	54.54 ± 8.99	58.87 ± 9.15	**<0.001**	0.65	**<0.001**
BMI (kg/m^2^)	23.14 ± 3.20	23.90 ± 3.52	24.89 ± 3.91	**0.03**	**<0.001**	**0.004**
Underweight (< 18.5 kg/m^2^)	10 (6.8%)	10 (3.1%)	8 (4.2%)	0.06	**<0.001**	**0.003**
Normal weight (18.5–22.9 kg/m^2^)	60 (40.8%)	135 (41.9%)	50 (26.3%)			
Overweight (23–24.9 kg/m^2^)	38 (25.9%)	63 (19.6%)	39 (20.5%)			
Obese (≥ 25 kg/m^2^)	39 (26.5%)	114 (35.4%)	93 (48.9%)			
Waist circumference (cm)	85.17 ± 7.96	83.14 ± 9.02	87.84 ± 9.92	**0.02**	**0.01**	**<0.001**
Systolic BP (mmHg)	123.57 ± 18.17	119.50 ± 15.62	125.50 ± 15.49	**0.02**	0.29	**<0.001**
Diastolic BP (mmHg)	70.99 ± 11.92	70.23 ± 10.56	73.25 ± 11.93	0.49	0.09	**0.004**
FPG (mmol/L)	5.82 ± 0.27	5.1 ± 0.28	5.93 ± 0.33	**<0.001**	**0.001**	**<0.001**
HbA_1c_ (mmol/mol)	34.90 ± 2.58	40.72 ± 1.94	41.67 ± 2.39	**<0.001**	**<0.001**	**<0.001**
Cr (mmol/L)	0.07 ± 0.02	0.07 ± 0.02	0.07 ± 0.02	0.62	0.31	0.06
TC (mmol/L)	5.45 ± 1.03	5.61 ± 0.96	5.65 ± 0.86	0.11	0.06	0.63
TG (mmol/L)	1.25 ± 0.64	1.27 ± 0.95	1.45 ± 0.78	0.89	**0.01**	**0.03**
HDL (mmol/L)	1.57 ± 0.46	1.62 ± 0.44	1.53 ± 0.43	0.29	0.33	**0.02**
LDL (mmol/L)	3.33 ± 0.96	3.42 ± 0.89	3.48 ± 0.82	0.31	0.12	0.45

**Notes**: Data presented as number and percentage or mean plus/minus standard deviation. Bold *P* values indicate statistically significant. ^a^Isolated IFG vs normal FPG with HbA_1c_ 38.8–46.4 mmol/mol. ^b^Isolated IFG vs IFG with HbA_1c_ 39–46 mmol/mol. ^c^Normal FPG with HbA_1c_ 38.8–46.4 mmol/mol vs IFG with HbA_1c_ 38.8–46.4 mmol/mol.

**Abbreviations**: PreDM, prediabetes mellitus; IFG, impaired fasting glucose; FPG, fasting plasma glucose; HbA1c, glycated hemoglobin; BMI, body mass index; BP, blood pressure; Cr, creatinine; TC, total cholesterol; TG, triglycerides; HDL, high-density lipoprotein cholesterol; LDL, low-density lipoprotein cholesterol.

### Comparison of Clinical Characteristics and Laboratory Parameters Between PreDM Participants with and without MetS

PreDM participants without MetS were significantly younger (56.15 ± 9.42 vs 57.91 ± 9.16 years, *P* = 0.03) and were more female (*P* = 0.31). BMI, waist circumference, systolic BP, and diastolic BP were all significantly higher in PreDM with MetS compared to PreDM without MetS (all *P* < 0.001). FPG and HbA_1c_ level were significantly higher but HDL cholesterol level was significantly lower in PreDM with MetS than in PreDM without MetS (all *P* < 0.001) (Table 3).

**Table 3:** Demographic, Clinical, and Laboratory Characteristics Compared Between PreDM Participants Without and with MetS

Characteristics	PreDM Without MetS (n = 471)	PreDM with MetS (n = 188)	*P*
Female gender	320 (67.9%)	120 (63.83%)	0.31
Age at survey (years)	56.15 ± 9.42	57.91 ± 9.16	**0.03**
BMI (kg/m^2^)	23.28 ± 3.40	25.86 ± 3.50	**<0.001**
Underweight (< 18.5 kg/m^2^)	28 (5.9%)	0 (0.0%)	**<0.001**
Normal weight (18.5–22.9 kg/m^2^)	210 (44.6%)	35 (18.6%)	
Overweight (23–24.9 kg/m^2^)	92 (19.5%)	48 (25.5%)	
Obese (≥ 25 kg/m^2^)	141 (29.9%)	105 (55.9%)	
Waist circumference (cm)	82.42 ± 8.63	91.27 ± 7.71	**<0.001**
Systolic BP (mmHg)	118.25 ± 14.51	131.88 ± 16.76	**<0.001**
Diastolic BP (mmHg)	68.86 ± 10.20	77.30 ± 11.81	**<0.001**
FPG (mmol/L)	5.38 ± 0.46	5.81 ± 0.43	**<0.001**
HbA_1c_ (mmol/mol)	39.71 ± 3.10	39.67 ± 4.13	0.91
Cr (mmol/L)	0.07 ± 0.02	0.07 ± 0.02	0.41
TC (mmol/L)	5.59 ± 0.92	5.56 ± 1.03	0.71
TG (mmol/L)	1.07 ± 0.49	1.92 ± 1.17	**<0.001**
HDL (mmol/L)	1.69 ± 0.43	1.3 ± 0.35	**<0.001**
LDL (mmol/L)	3.41 ± 0.86	3.43 ± 0.97	0.84

**Notes**: Data presented as number and percentage or mean plus/minus standard deviation. Bold *P* values indicate statistically significant.

**Abbreviations**: PreDM, prediabetes mellitus; MetS, metabolic syndrome; BMI, body mass index; BP, blood pressure; FPG, fasting plasma glucose; HbA_1c_, glycated hemoglobin; Cr, creatinine; TC, total cholesterol; TG, triglycerides; HDL, high-density lipoprotein cholesterol; LDL, low-density lipoprotein cholesterol.

### Association Among Clinical Parameters, MetS Score, MetS Criteria and Risk of PreDM

Univariate and multivariate analyses for factors independently associated with PreDM were shown in Table 4. After adjusting for age, gender, and BMI, female gender was found to be an independent protective factor against developing PreDM (OR: 0.58, 95% CI: 0.44–0.76; *P* < 0.001). In contrast, a MetS score of 3 (OR: 2.98, 95% CI: 1.99–4.49; *P* < 0.001); a MetS score of 4 or 5 (OR: 9.02, 95% CI: 4.03–20.18; *P* < 0.001); obese status (OR: 1.8, 95% CI: 1.07–3.04; *P* = 0.03); and low HDL cholesterol level (OR: 1.42, 95% CI: 1.02–1.96; *P* = 0.04) were all found to be independently associated with higher risk of developing PreDM. There was no association between age, blood pressure, waist circumference, triglyceride level, and the risk of PreDM.

**Table 4:** Univariate and Multivariate Analysis for Factors Independently Associated with Prediabetes

Factors	PreDM	Normoglycemia	Unadjusted	*P*	Adjusted OR^a^	*P*
	(n = 659)	(n=541)	OR (95% CI)		OR (95% CI)	
**By MetS risk score**						
Score 0–2	71.5%	92.1%	1.00 (reference)	–	1.00 (reference)	–
Score 3	17.8%	6.7%	3.44 (2.32–5.10)	**<0.001**	2.98 (1.99–4.49)	**<0.001**
Score 4–5	10.8%	1.3%	10.72 (4.88–23.55)	**<0.001**	9.02 (4.03–20.18)	**<0.001**
**By age**						
Age < 48 years	17.6%	31.2%	1.00 (reference)	–	1.00 (reference)	–
Age 48–61 years	53%	51.2%	1.84 (1.38–2.44)	**<0.001**	1.15 (0.74–1.79)	0.54
Age > 61 years	29.4%	17.6%	2.98 (2.12–4.18)	**<0.001**	1.04 (0.47–2.29)	0.93
**By gender**						
Male	33.2%	21.1%	1.00 (reference)	–	1.00 (reference)	–
Female	66.8%	78.9%	0.54 (0.41–0.70)	**<0.001**	0.58 (0.44–0.76)	**<0.001**
**By BMI**						
Underweight and normal weight	41.4%	50.6%	1.00 (reference)	–	1.00 (reference)	–
Overweight	21.2%	24.2%	1.07 (0.80–1.44)	0.64	0.97 (0.67–1.39)	0.86
Obese	37.3%	25.1%	1.82 (1.39–2.37)	**<0.001**	1.80 (1.07–3.04)	**0.03**
**By MetS criteria**						
**Waist circumference**						
Normal waist circumference	41.0%	45.3%	1.00 (reference)	–	1.00 (reference)	–
Abdominal obesity	59.0%	54.7%	1.19 (0.95–1.50)	0.13	0.94 (0.70–1.26)	0.68
**Serum triglyceride**						
Normal triglyceride	79.2%	85.8%	1.00 (reference)	–	1.00 (reference)	–
Hypertriglyceridemia	20.8%	14.2%	1.58 (1.17–2.15)	**0.003**	1.33 (0.97–1.84)	0.08
**Serum HDL cholesterol**						
Normal HDL cholesterol	80.7%	85.8%	1.00 (reference)	–	1.00 (reference)	–
Low HDL cholesterol	19.3%	14.2%	1.44 (1.06–1.96)	**0.02**	1.42 (1.02–1.96)	**0.04**
**Blood pressure**						
Normal blood pressure	67.5%	75.2%	1.00 (reference)	–	1.00 (reference)	–
High blood pressure	32.5%	24.8%	1.46 (1.13–1.88)	**0.004**	1.10 (0.84–1.45)	0.49

**Notes**: Bold *P* values indicate statistically significant. ^a^Unconditional logistic regression analysis was adjusted for age, gender, and BMI. Baseline analysis was normoglycemia participants.

**Abbreviations**: PreDM, prediabetes mellitus; OR, odds ratio; CI, confidence interval; MetS, metabolic syndrome; BMI, body mass index; HDL, high-density lipoprotein cholesterol.

## Discussion

This is the first study in a large well-characterized cohort to assess the prevalence of PreDM and MetS in seemingly healthy Thais. Demographic, clinical, and laboratory parameters were also compared between PreDM with and without MetS. Interestingly, a meaningful proportion of PreDM (54.3%) and diabetes (1.07%) was demonstrated, which highlights the importance of routine health check-ups. Moreover, serum creatinine was lower in diabetes than in PreDM. The Japan Epidemiology Collaboration on Occupational Health Study reported low serum creatinine to be significantly associated with an increased risk of diabetes.^19^

Participants with isolated IFG had lower HDL cholesterol levels than PreDM participants with normal FPG and HbA_1c_ 38.8–46.4 mmol/mol. This finding is in agreement with Telles S et al who reported a negative association between FPG and HDL cholesterol levels, but a positive correlation between FPG and waist circumference in healthy obese adults.^20^ Elevated hepatic insulin resistance is a typical finding in isolated IFG, with almost normal skeletal muscle sensitivity.^21^ Drew et al showed that high-density lipoprotein could modulate glucose metabolism by promoting insulin secretion and by activating AMP-activated protein kinase in skeletal muscles. Thus, higher HDL cholesterol levels were linked with lower blood glucose levels.^22^

A highlight of this study is that PreDM with HbA_1c_ 38.8–46.4 mmol/mol, but normal FPG was examined. To the best of our knowledge, no study has investigated the clinical characteristics and metabolic profiles of this PreDM subgroup. The findings that they were younger, more likely to be female, and had the lowest systolic blood pressure and waist circumference of the three investigated PreDM subgroups suggests diverse pathogenesis. IFG with HbA_1c_ 38.8–46.4 mmol/mol subgroup participants were the oldest and had the worst metabolic features compared to the other two PreDM subgroups. The combination of HbA_1c_ and FPG levels was reported to improve risk prediction for developing diabetes.^23^

Our study also showed that PreDM subjects with MetS accounted for 28.5% of all PreDM participants. They were older and have features suggestive of greater insulin resistance, such as higher BMI, blood pressure, waist circumference, and triglyceride, in addition to lower HDL cholesterol levels. Diamantopoulos and colleagues showed that PreDM and MetS were not identical^16^ and a study by Ghachem demonstrated that different categories of PreDM were associated with different features of MetS such as BMI, waist circumference, lipid parameters, and CRP level.^24^ Both studies were done in the Caucasian population and the MetS score was not considered account. To the best of our knowledge, there was no such study in Thais. In addition, our study also showed that the higher the MetS score, the greater risk of PreDM. These findings indicated that MetS is relatively common in PreDM patients. Obesity and low HDL cholesterol level were major determinants of PreDM in this study. A study by Sofer et al demonstrated a strong correlation between circulating insulin-degrading enzyme (IDE) levels and circulating levels of triglycerides, insulin, and C-peptide but an inverse correlation with HDL cholesterol. Serum IDE levels were higher in MetS subjects than in controls.^25^ As a result, circulating IDE may serve as a tool to identify subjects with abnormal insulin metabolism, and possibly those with MetS and PreDM.

Multivariate logistic regression analysis with adjustment for age, BMI, and gender revealed that males, with higher BMI, MetS score, and lower HDL cholesterol levels were independent predictors of PreDM. Obesity was reported to significantly influence the development of PreDM in the Northeast Chinese population.^26^ A higher proportion of PreDM was also found among men in the US population.^27^ Low HDL cholesterol level in patients with PreDM and MetS is a risk factor for heart disease, stroke,^28^ and T2D.^29^ Previous study reported that HDL cholesterol could enhance skeletal muscle absorption of glucose^22^ and that it promoted insulin secretion from pancreatic beta cells. Thus, low HDL cholesterol levels may be associated with dysglycemia.^30^

## Limitations

This cross-sectional study had some limitations. First, it was conducted at a university-based tertiary care hospital, these findings may not represent the prevalence of PreDM and MetS in other locations and care settings in Thailand. A national multicenter study is, therefore, needed. Second – OGTT, if performed, may yield a better classification of PreDM, which would include impaired glucose tolerance (IGT) whether isolated or in combination with IFG and/or HbA_1c_ 38.8–46.4 mmol/mol. The age and sex distribution of Isolated IFG and isolated IGT were different and the prevalence of both conditions increased with advancing age.^31^ IGT is more frequent in women while isolated IFG and a combination of IFG and IGT were more common in men.^32^ Moreover, the underlying pathophysiology of both conditions was considerably different. Subjects with isolated IFG showed a defect in the first phase and early-phase insulin response to glucose stimulation with predominantly hepatic insulin resistance. In contrast, individuals with isolated IGT exhibited moderate to severe muscle insulin resistance and markedly deficit late-phase insulin secretion.^33^ However, since the reproducibility of the test is poor and is relatively time-consuming, OGTT is not suitable in a health check-up setting. Third, it is possible that distinct PreDM subgroups have diverse characteristics, different relationships with MetS, and different rates of progression to diabetes and the development of cardiovascular disease. Hence, long-term follow-up is needed to fully understand the natural history of various PreDM subgroups. A prospective study to assess whether lifestyle modification and/or certain medications may prevent or delay the development of T2D and cardiovascular disease in this population is also warranted. Forth, there is emerging evidence that nonalcoholic fatty liver disease (NAFLD) is relatively common in PreDM. A study by Vesa et al^34^ revealed that the prevalence of NAFLD was 48.25% in PreDM. Moreover, NAFLD was also associated with an increased risk of atrial fibrillation and chronic kidney disease.^35^,^36^ Since measurement of transaminase enzyme level/ultrasound abdomen was not done in our study, the information regarding NAFLD is not available.

## Conclusion

The prevalence of glucose intolerance (PreDM or diabetes) and MetS was relatively high in seemingly healthy persons in this study. Since both PreDM and MetS increase the risk of diabetes and cardiovascular disease, early detection via a health check-up at an appropriate time interval is essential. In addition, PreDM diagnosed by different glycemic parameters (FPG and HbA_1c_) demonstrated different clinical characteristics and laboratory parameters, which may indicate different pathogenesis, and that the risk of diabetes and cardiovascular disease may vary. Moreover, participants with PreDM and MetS demonstrated features suggestive of insulin resistance phenotypes. Future studies examining the development and prevention of diabetes and cardiovascular diseases in these particular clusters are needed.
